# Sollerman Hand Function Sub-Test “Write with a Pen”: A Computer-Vision-Based Approach in Rehabilitation Assessment

**DOI:** 10.3390/s23146449

**Published:** 2023-07-17

**Authors:** Orestis N. Zestas, Nikolaos D. Tselikas

**Affiliations:** Communication Networks and Applications Laboratory (CNALab), Department of Informatics and Telecommunications, University of Peloponnese, 221 00 Tripoli, Greece; o.zestas@go.uop.gr

**Keywords:** sollerman hand function test, computer vision, rehabilitation assessment, upper-limb rehabilitation, fine motor skills, shape detection

## Abstract

Impaired hand function is one of the most frequently persistent consequences of stroke. Throughout the rehabilitation process, physicians consistently monitor patients and perform kinematic evaluations in order to assess their overall progress in motor recovery. The Sollerman Hand Function Test (SHT) is a valuable assessment tool used to evaluate a patient’s capacity to engage in daily activities. It holds great importance in the field of medicine as it aids in the assessment of treatment effectiveness. Nevertheless, the requirement for a therapist’s physical presence and the use of specialized materials make the test time-consuming and reliant on clinic availability. In this paper, we propose a computer-vision-based approach to the “Write with a pen” sub-test, originally included in the SHT. Our implementation does not require extra hardware equipment and is able to run on lower-end hardware specifications, using a single RGB camera. We have incorporated all the original test’s guidelines and scoring methods into our application, additionally providing an accurate hand spasticity evaluator. After briefly presenting the current research approaches, we analyze and demonstrate our application, as well as discuss some issues and limitations. Lastly, we share some preliminary findings from real-world application usage conducted at the University campus and outline our future plans.

## 1. Introduction

The annual number of strokes and deaths due to stroke has increased substantially in the last three decades. Globally, stroke remained the second-leading cause of death and the third-leading cause of death and disability combined in 2019 [1]. Approximately 80% of individuals who have experienced a stroke encounter prevalent dysfunctions such as upper limb hemiplegia and impaired finger dexterity [2,3]. Therefore, healthcare professionals advocate for structured motor assessments and regular monitoring of hand function in individuals who have experienced a stroke. This approach allows clinicians to assess the patients’ manual dexterity in various daily activities, ensuring a comprehensive evaluation of their recovery progress [4]. In addition, the rise in stroke incidence leads to increasing needs for neurorehabilitation and for new effective treatment methods using state-of-the art techniques and technologies, such as computer vision applications. The development of such implementations may help clinicians objectively measure a patient’s performance using the automatic computation of quantitative measures, therefore reducing inter-rater subjectivity in the classical evaluation. This procedure may also be used in home-based rehabilitation practices, where patients could self-rehabilitate and assess their cognitive and motor improvements using validated virtual analyses.

Evaluating hand function is a crucial aspect of understanding the progression of disorders and gauging the effectiveness of treatments. The ability to accurately assess hand function and grip performance in daily activities is vital for therapists in evaluating patients’ hand performance and devising appropriate treatment strategies. The Sollerman Hand Function Test (SHT) is a standardized and validated assessment tool and a reliable method to measure overall hand function [5,6]. By employing the SHT, therapists can ensure precise evaluation and enhance the development of tailored treatment plans for patients. The SHT comprises a set of 20 activities of daily living (ADL) tasks, each involving specific grip patterns. The test evaluates the time taken to complete each task and the quality of the performed movements [5]. While the SHT is known for its high accuracy, one significant limitation is its dependence on the constant presence of a doctor or therapist, as well as its reliance on clinic-based administration.

In this paper, we examine a computerized rendition of the “Write with a pen” ADL task, originally part of the Sollerman Hand Function Test. Our solution integrates the fundamental aspects of the real-world test into a computer-vision-based approach, leveraging the capabilities of the MediaPipe Hand Tracking solution to accurately detect and track the position and motion of the user’s hand and fingers. Our proposed implementation addresses the limitations found in the original SHT sub-test, and consolidates the whole functionality into a single, efficient application. This also eliminates the need for additional equipment, such as smart gloves and/or Virtual Reality (VR) headsets, while operating seamlessly on a lower-end hardware range PC, with a single RGB camera (either external or built-in) connected.

The remainder of this paper is organized as follows. Section 2 provides a study of the current research approaches and a literature review concerning virtual implementations of upper-limb rehabilitation practices, such as the SHT. Section 3 examines and showcases our computer-vision implementation of the SHT sub-test, while its scoring system and spasticity evaluator are presented in Section 4. In Section 5, we discuss some technical issues and limitations of the application. Finally, the paper is concluded in Section 6, where some future work that can be completed in order to further develop our system is also presented.

## 2. Current Research and Approaches

Hand rehabilitation constitutes an essential component in the process of restoring hand and finger health as well as functionality. The prevailing rehabilitation protocols emphasize the significance of motor learning, as it provides a fundamental framework for facilitating motor recovery [7]. The research community has proposed quite a few approaches to digital versions of hand rehabilitation exercises and tests, such as the development of digital Box and Block Tests (BBTs) [8,9,10], which is one of the most used and recommended tools to evaluate unilateral manual dexterity.

At the time of writing, the literature contains only two studies that have developed working implementations of digital SHTs. One approach proposes a web version of the “Open/Close Zip” sub-test included in the SHT [11], while the other offers native desktop implementations of two SHT evaluation tests [12]. Other research studies suggest similar hand activities that aim to enhance the quality of life for individuals in their everyday tasks. For example, a study suggests the design of a touch-less user interface where users with partial physical disabilities can interact with electronic devices by performing natural gestures and tracing the contours of geometric and non-geometric shapes within the field of view of a motion sensor (Leap Motion controller-based methodology) [13].

Furthermore, a research study recommends a hand-writing rehabilitation exercise in a haptic virtual environment [14], where patients can recover their writing skills. This application can be employed to steer the user’s hand along a designated path by utilizing real-time guidance force. To simulate a virtual pen for writing English characters, a haptic device known as PHANTOM Premium 1.0 is utilized, serving as both a writing tool and a means to provide force feedback for guidance purposes. However, while the results are very promising, the use of very specific additional hardware is required.

Finally, in addition to the extensive research conducted on upper-limb motion analysis, the scientific community has also put forth several methodologies for digital analysis of lower-limb motion. These approaches often involve the integration of exoskeleton technology alongside bio-electronic devices, enabling the acquisition of valuable biological and kinematic data pertaining to the lower-limb [15].

## 3. Computer Vision Approach Designed for Rehabilitation

Our Computer Vision Sollerman Writing Test (CV-SWT) aims to provide a fast, accurate, but lightweight upper-limb rehabilitation exercise while following the original test’s guidelines. The application does not require any kind of specialized computer peripherals or substantial computing power, other than a single RGB camera. From our testing, the system performs in its optimal state when the camera is positioned about 50 cm from the patient’s hand and there is enough natural light present in the testing environment.

### 3.1. Modules

CV-SWT has been developed with Python 3.10, using OpenCV [16] and MediaPipe’s Hand solution [17], two robust and modern computer vision libraries. The OpenCV library processes each individual frame and generates the necessary graphical elements, while MediaPipe Hands ensures swift and accurate predictions of human hand gestures and finger positions.

Our system’s development can be divided into four essential segments: an input shape detection algorithm, the creation and modeling of the virtual environment, tracking the patient’s hand, and finally the application of the exercise’s necessary operational logic.

### 3.2. Input Shape Detection Algorithm

In order to create the interactive computer vision environment, the system must first initialize a target shape, which will be tracked throughout the whole duration of the test. This process needs to be dynamic so as to enable the physician to create a shape of his own choosing, depending on the patient’s needs and current rehabilitation state. Thus, a shape detection algorithm is implemented, which processes an input image file containing the desired shape and creates an identical digital version that is interact-able. This also enables us to accurately check the shape percentage that has been successfully drawn by the patient as well as calculate an estimate of the hand’s standard deviation from the shape (spasticity), both of which will be discussed in the coming sections.

**Figure 1:**
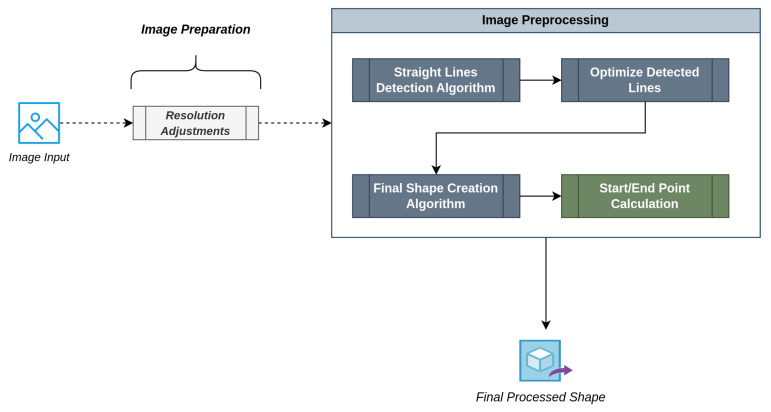
Shape Detection Algorithm Overview.

A high-level approach to our shape detection algorithm is depicted in Figure 1. As aforementioned, the system initially reads an input image file containing a primal version of the desired shape. Note that this image can either be a picture of a hand-drawn shape by the physician or a virtually created one using any image manipulation program. The only constraints that need to be taken into account are:

- The image must be in black and white (black shape lines, white background).
- The final image resolution must be 512 by 512 pixels.
- The shape must only contain straight lines with no curves.

After some basic image resolution adjustments, the algorithm is ready to start processing the input image and detect the containing shape, which will be later utilized by our implementation.

**Figure 2:**
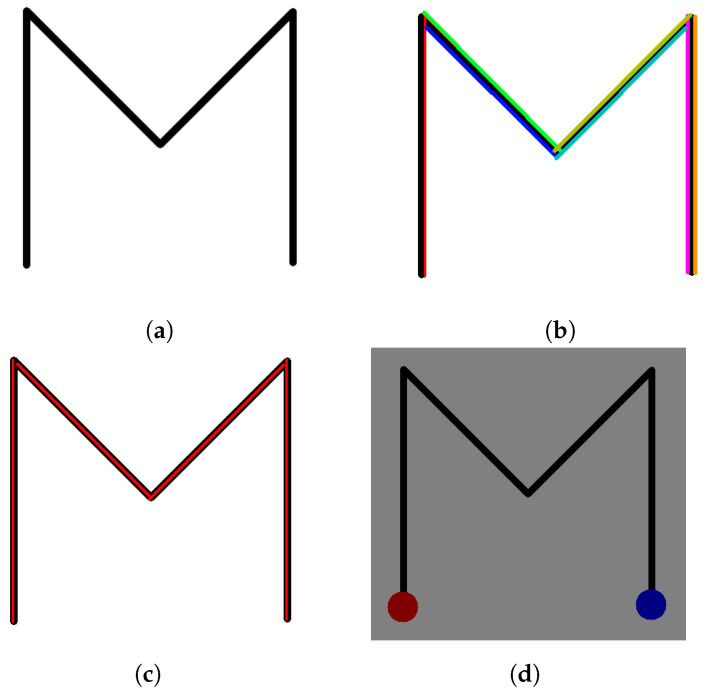
Example Output of the Line Detector. (**a**) Original Image; (**b**) Output Shape With Detected Lines; (**c**) Final Detected Lines After Optimization; (**d**) Example Shape Start/End Point.

Starting off, the system applies the Canny edge detection algorithm [18] (developed by John F. Canny in 1986), which is a widely utilized technique in computer vision and image processing for detecting edges in digital images. When applying the algorithm, we set the first threshold for the hysteresis procedure to 50, the second threshold to 150, and the aperture size for the Sobel operator (to find image gradients) is set to 3. The aforementioned values, determined through empirical analysis and thorough testing, produce the best possible results for an image containing only black and white colors. In essence, the hysteresis thresholding determines the true edges by applying two threshold values, *minVal* and *maxVal*. Edges with intensity gradients above *maxVal* are confirmed as edges, while those below *minVal* are identified as non-edges and discarded. Edges lying between these two thresholds are assessed based on their connectivity to determine whether they should be classified as edges or non-edges. If they are connected to pixels identified as “sure-edges”, they are recognized as part of the edge structure. Conversely, if they lack such connectivity, they are also discarded. The next step is to extract all straight lines from the image. Here, the algorithm applies the Hough Line Transform, where it utilizes the previously created edge map from the Canny edge detector and accumulates the presence of edge pixels in the Hough space, where each pixel corresponds to a potential line parameter. In our implementation, we applied the probabilistic version of the Hough Lines Transform [19], which considers only a subset of the edge points, resulting in a significant reduction in computational complexity. This approach randomly selects edge points rather than searching for lines across the entire parameter space and then performs line fittings on these selected points. By employing a voting scheme, the most probable lines in the image are determined by the algorithm and are represented as line segments with their corresponding start and end points. Figure 2 demonstrates an example of line detection that occurs from the letter “*M*”. Figure 2a shows the original image, whereas Figure 2b shows the algorithm’s output. While the algorithm does a good job of detecting the overall shape, the end result contains more lines than expected. In this example, the letter “*M*” is drawn with four straight lines, though the algorithm has calculated seven, as depicted with different colors in Figure 2b. This can cause various problems in the later stages of the application, especially regarding collision checking with the patient’s fingers. Thus, further optimization of the detection process is needed in order to calculate the exact number of lines required for the final shape and maintain their correct overlay on the original shape image. To do this, the system initially iterates through an array of all the detected line points and calculates the mean value of their coordinates as well as their respective line segment’s slope. This information helps the algorithm decide if each line generated by the Hough Lines Transform has a duplicate, or not. If a duplicate line is found based on the distance and slope difference, then the system automatically discards it and adjusts the respective line segment’s coordinates accordingly. As illustrated in Figure 2c, the lines detected by the system after optimization perfectly match the original input shape.

As a final procedure, the system determines where the shape tracing starts and where it ends. Note that this process differs for both open and closed shapes. In the previous example, the letter “*M*” is considered an open shape since it does not enclose an area and its lines do not connect back to their beginning. On the other hand, a closed shape refers to a shape that forms a complete loop or enclosure, such as a triangle. Subsequently, if the input image contains a closed shape, then the starting point matches the end point, creating a loop. Additionally, in the case of an open shape, the algorithm considers the bottom leftmost point as the start and the bottom rightmost point as the end of the tracing procedure, as shown in Figure 2d.

### 3.3. Environment Modeling

The next step of our implementation involves setting up the computer vision environment of the CV-SWT test. Since an interactive digital shape was constructed in the previous stage, it is available for utilization by the frame creation process. This procedure initially connects to a single RGB camera on the host PC and begins by capturing frames repetitively. Note that, for our testing, we used a 60 fps (frames per second) camera with a resolution of 720p (1280 × 720), which yields optimal results. Once a single frame is successfully captured, the image is flipped to better represent the patient’s hand movements, with the subsequent draw procedure of all the necessary persistent graphical elements, such as the timer and title. Before continuing, the system also projects the original input shape on top of the captured camera frame, scaled to match the application’s running resolution. The scaling process depends on the host PC display and camera resolutions.

Following the successful frame pre-processing procedure, the detected line segments forming the digital shape are drawn in the image based on the respective arrays of calculated edges. Moreover, the tracing’s start and end points are also projected on top of the shape, colored red and blue, respectively.

### 3.4. Hand Detection & Operation Logic

In order to accommodate an accurate hand pose detection logic, we utilized Google’s MediaPipe, which employs a multi-stage pipeline architecture for the detection of human hands. The process begins by feeding the previously captured frames into the camera into a series of computer vision and machine learning modules for analysis. In the first stage, MediaPipe applies a hand detection model to identify potential hand regions within the frame, which is trained to recognize hand-like features and generate bounding boxes around the detected hands. Once the hand regions are identified, the pipeline moves to the next stage, which involves the application of a hand landmark model that analyzes the hand regions and predicts the positions of various key landmarks such as fingertips, palm centers, and joints. The calculated landmarks provide crucial information about the hand’s pose and shape that will be utilized by the system in order to apply the test’s core operations. By considering the previous frames’ (iteration) bounding box and applying various procedures such as Kalman filtering or moving average, MediaPipe enhances the stability and accuracy of hand tracking, reducing jitter or inconsistencies in the output. In essence, this means that the hand detector is not applied to every successful frame capture. Instead, it is only applied to the initial frame or when the hand is completely lost from the box. In addition, to further refine the hand pose estimation, the algorithm also utilizes temporal filtering and smoothing techniques. For our CV-SWT implementation, only one hand needs to be recognized and tracked throughout the whole process. If more than one hand is visible, then the first one to be detected is the one that the patient will work with, while the other is ignored.

As performed in the real-world scenario, the patient is initially instructed to perform the tripod pinch in order to emulate a writing behavior similar to using a pen. Furthermore, whilst holding the aforementioned grip, the index finger must be placed inside the red dot’s central area, indicating the beginning of the test. Once the patient has successfully initiated the tracing procedure, the goal is to completely draw the shape’s perimeter without losing the prescribed grip or drawing outside the predefined boundaries of the digital shape. With the aim of improving the test’s flexibility in accordance with the patient’s ongoing rehabilitation progress, the drawing point radius is configurable, so that it increases or decreases the digital boundary tolerance, providing a simple difficulty adjustment. As the radius of the drawing point increases, the test becomes increasingly effortless.

**Figure 3:**
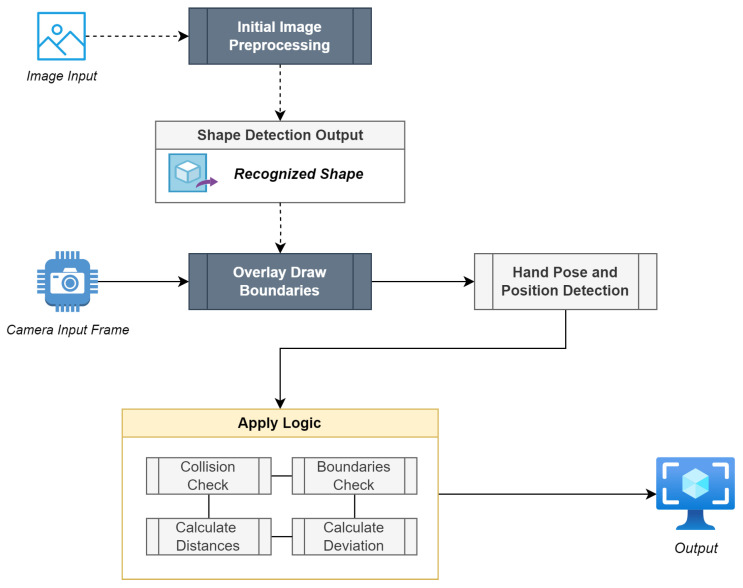
Operation Logic Overview.

**Figure 4:**
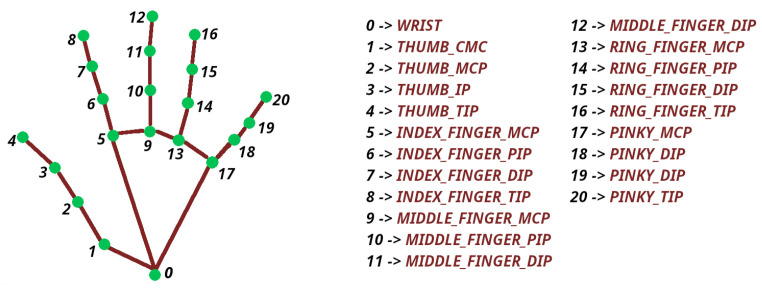
MediaPipe Hand Landmarks.

An overview of the test’s operation logic is illustrated in Figure 3. After successfully pre-processing every captured camera frame using the procedure described in Section 3.3, the system proceeds with the hand’s pose and position estimation. With the goal of ensuring that the patient’s grip is correct at all times (tripod pinch), the algorithm employs hand landmark checking, as provided by MediaPipe. The available hand landmarks are depicted in Figure 4. Since the tripod pinch requires the thumb, index, and middle fingers to be closed, the distance between them must be lower than a preset threshold, a number that is adjusted to work best when the hand is about 50 cm from the camera. Calculating finger distances is a process of applying the Pythagorean theorem to the triangle that is formed by the fingers themselves, where a single edge is considered the palm-to-fingertip distance. In this case, the participating fingers’ landmarks are 4 (*THUMB_TIP*), 8 (*INDEX_FINGER_TIP*), and 12 (*MIDDLE_FINGER_TIP*), where the Pythagorean theorem is applied a total of two times to compute the index-to-thumb as well as the middle-to-thumb distances.

Once the correct hand grip is ensured, the subsequent phase involves the hand’s position calculation relative to the camera. This is crucial to the application’s logic since it verifies that:

1. The initial positioning of the index finger is within the designated location of the red dot and,
2. The positioning of the index finger always resides within the computed shape boundaries, on every captured frame.

The first point requires a simple distance calculation, which is completed once at the test’s beginning, while the second employs collision checking repetitively. The process of validating a legal drawing position, which is defined as the *index finger position + the drawing radius*, is a two-step method. The system primarily checks if the current drawing point is situated amidst two points that form a single line of the shape, followed by an evaluation of collision with the same line’s general function. If the process discerns that each new calculated drawing point (once every frame) is legal, then the test continues and the point is drawn on the output image. On the other hand, if the drawing point resides outside the shape’s boundaries, the test fails immediately and has to be restarted.

As a conclusive step to validate the successful test’s completion, the system checks whether the index finger has reached its stopping point, which is different for open and closed shapes. As aforementioned, an open shape’s ending point is represented by a blue circle, whereas in closed shapes, it is represented by a red colored circle, which is also the trace’s beginning. In an additional safety step, the implementation also takes into account the calculated total length of the user’s drawing. If this value closely matches the digital shape’s total length, and the index finger is located within the vicinity of the ending point, then the test is considered completed.

### 3.5. Demonstration

**Figure 5:**
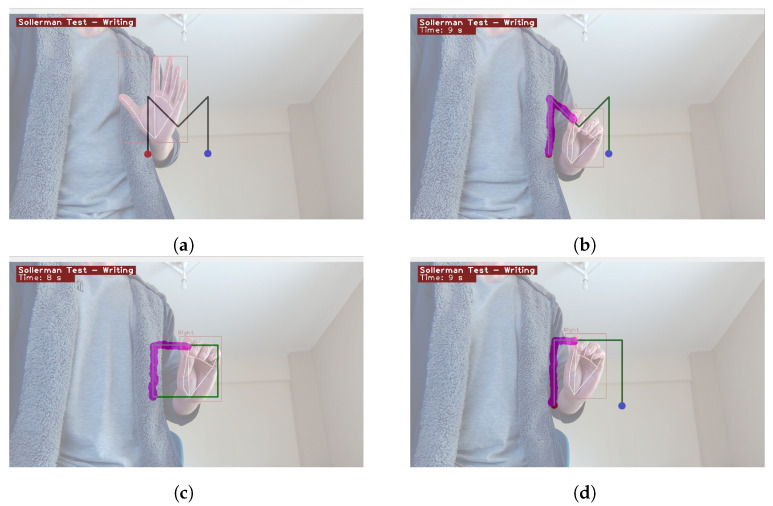
Example Application Demonstration. (**a**) Hand Detection; (**b**) Drawing of Letter “*M*”; (**c**) Drawing of a Rectangle; (**d**) Drawing of Greek Capital Letter “Π”. Red color indicates the starting point, while blue represents the ending point. Green lines indicate the shape’s path.

An example demonstration of the CV-SWT is illustrated in Figure 5. Figure 5a displays the idle state, where the test has not yet been initiated and only the hand detector is being applied on the frame. Figure 5b presents the test’s running state, where the patient is instructed to replicate the letter “*M*”. Furthermore, Figure 5c,d display different shapes, namely a rectangle and the Greek capital letter Π, respectively. Furthermore, the drawing point radius is set to its lowest possible value, which is 20 pixels. This makes the test more challenging while also improving its representation of the real-world scenario. If the patient is unable to successfully complete the test, the drawing point radius can be augmented to a maximum of 40 pixels, thereby facilitating a less demanding overall procedure.

From our testing, the CV-SWT can run on almost any PC with virtually no software or hardware requirements other than a working python3 installation and a single camera of any resolution. The application is also developed to be OS-independent and ships with all the necessary functionality bundled within a lightweight package. Though, to achieve optimal results, cameras with higher resolutions and frame rates are preferred so that they better emulate the user’s hand movements. Our CV-SWT implementation is open-sourced and available in [20].

## 4. Score and Hand Spasticity Evaluation

Each Sollerman sub-test is evaluated based on a rating scale ranging from 0 to 4 points, as elaborated in [5,6]. The grading process for evaluating the patient’s performance is outlined in Table 1 and is implemented virtually in order for this tool to be valid and reliable. Once the exercise has been completed, the system assigns a score to the patient, which comprises two key factors; time and distance drawn. Note that in the case of drawing out-of-boundaries or failing to trace beyond 1/3 of the shape, the assigned score in every instance is 0. Conversely, if the shape has been traced for a significant amount but not at its whole within 60 s, the attributed score is 1. In all other cases, i.e., when the shape has been traced completely with success, the final appointed score is calculated based on the completion time, as depicted in Table 1.

Alongside the formerly mentioned grading process, the system additionally integrates a hand spasticity assessment module, thereby aiding the physician in conducting a more precise evaluation of the patient’s current rehabilitation status. To facilitate this functionality, the system computes the signed distance between the drawing point’s center and the corresponding line each time collision checking is applied. This data is subsequently stored in an array of doubles, and each new calculation is appended after every successfully processed camera frame. The hand’s spasticity is defined as the mean value of all stored distances, i.e., the hand’s mean deviation from the digital shape. Upon the completion of the test, the achieved score and hand spasticity measurements are recorded and stored in an output CSV file, ensuring their subsequent scrutiny by the attending physician. This meticulous record-keeping practice enables the healthcare professional to thoroughly review and analyze the collected data, contributing to a more comprehensive evaluation of the patient’s performance and spasticity levels throughout the rehabilitation process.

## 5. Discussion and Further Issues

Virtual exercises or evaluation tests for upper-limb rehabilitation practices are challenging endeavors that necessitate thorough validation. Our proposed application incorporates various advanced computer vision capabilities in order to effectively implement a renowned hand function assessment tool. The CV-SWT provides the physician not only with a solid means of assessing the current upper-limb dexterity of a patient, but an accurate virtual tool for distant monitoring, as well. The latter is quite important, since granting patients the autonomy to manage their rehabilitation activities in a self-directed manner not only fosters a sense of ownership and personal agency but also facilitates a more tailored and flexible approach to their therapeutic journey. In addition, the patient is encouraged to engage in modern neurorehabilitation techniques, fostering their motivation to actively participate in the rehabilitation process.

Furthermore, it is also important to ensure that the test’s accuracy and results closely match those of the real-world scenario, as conducted under the supervision of a physician. Thus, it is crucial to capture and process each frame in near real-time (even when running on lower-end hardware) while ensuring the accurate implementation of the scoring system. Our computer vision exercise does not require fast reaction times (such that accuracy is not sacrificed for speed), and has yielded some promising results when running on mid-range hardware while using MediaPipe in combination with the OpenCV library (30–40 ms process time per frame).

**Figure 6:**
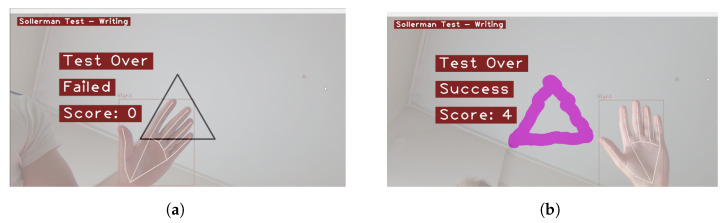
Testing CV-SWT on the University Campus of the UoP. (**a**) Assigned score: 0 (failed completion); (**b**) Assigned score: 4 (successful completion under 20 s). The color pink indicates the drawn shape path by the patient.

In order to thoroughly evaluate the effectiveness and clinical viability of our computer vision approach, we conducted a comprehensive testing phase involving a wide range of participants, including males and females with various dominant hand settings. This study took place at the university campus of the University of Peloponnese (UoP), where we engaged with both students and teachers who possess healthy hand function. Example test outcomes are presented in Figure 6. By involving individuals from various backgrounds and age groups, we aimed to gather diverse perspectives and ensure the robustness of our findings. The results obtained from this extensive testing not only provide compelling evidence of the validity of our approach but also highlight its potential as a valuable tool for healthcare professionals in clinical settings.

During the implementation phase of our application, we encountered one notable technical challenge that pertains to the accuracy of hand pose prediction, a crucial aspect of our CV-SWT. The detection of the patient’s hand is susceptible to various environmental factors, including but not limited to room lighting conditions, diverse backgrounds, and variations in the user’s hand appearance. Consequently, there were instances where the finger readings were not entirely precise, rendering the successful execution of the test more difficult. To mitigate this issue, it is preferable to conduct all the testing in a naturally lit room, as this tends to yield better results. However, in situations where natural lighting is unavailable, the use of warm, non-white illumination lamps positioned in such a manner that they are away from direct camera exposure can provide satisfactory performance, even in dimly lit settings.

Additionally, a notable drawback of our implementation lies in the limited capability of the shape detection algorithm, which focuses exclusively on identifying shapes composed of straight lines. This deliberate design choice is motivated by the objective of minimizing the overall complexity of the application. By restricting the algorithm to straight-line shapes, we aim to streamline the processing and analysis tasks, ensuring efficient performance and reducing computational overhead. However, this simplification may result in the algorithm overlooking more intricate shapes that involve curved or non-linear elements, compromising its ability to comprehensively detect and analyze such complex geometries.

## 6. Conclusions and Further Development

We have presented a computer-vision-based SHT writing test that serves both as a tool for assessing hand function and as an exercise platform for enhancing fine motor skills. This approach requires no extra specialized equipment, as it only necessitates a PC with no high-end specifications and a single RGB camera. The application has the capability to accept an input in the form of an abstract shape that solely consists of straight lines. This shape is then processed by a shape detection algorithm and transformed into a digital interactive form, which the patient is instructed to trace. We have also employed a reliable method for accurately detecting and tracking the patient’s hand and fingers, ensuring optimal performance even on lower-end hardware configurations. Additionally, we have seamlessly integrated the scoring system of the SHT writing sub-test into our virtual implementation, along with a valid hand spasticity evaluator, providing a comprehensive and standardized assessment of hand functionality.

In the near future, we plan to deploy this advanced computer-vision approach as a credible and dependable solution, designed specifically for utilization by physiotherapists and other professionals in the field of rehabilitation. Our intention is to provide a valuable tool that can be easily implemented by experts to enhance their practice and improve the quality of care provided to patients. Additionally, our future plans involve the implementation of a Clinical Decision Support System (CDSS), which aims to enhance the capabilities of rehabilitation experts in monitoring and assessing patients. This CDSS will serve as a robust tool, providing valuable insights and support throughout the rehabilitation process. Lastly, we aim to incorporate a shape detection algorithm and operational logic that encompass various types of shapes, not limited to those composed exclusively of straight lines, while maintaining high processing speeds without any reduction in performance.

## Figures and Tables

**Table 1:** SHT Sub-test Performance Grading.

Score	Performance
0	The patient could not carry out the task
1	The task was partially performed within 60 s
2	The task was completed, but with great difficulty, or the task was completed within 60 s but not less than 40 s
3	The task was completed, but with slight difficulty, or the task was completed within 40 s but not less than 20 s
4	The task was carried out without any difficulty within 20 s

## Data Availability

Not applicable.

## Abbreviations

The following abbreviations are used in this manuscript:

ADL	Activities of Daily Living
CDSS	Clinical Decision Support System
CSV	Comma-Seperated Values
CV-SWT	Computer Vision Sollerman Writing Test
OS	Operating System
PC	Personal Computer
RGB	Red Green Blue
SHT	Sollerman Hand Function Test
UoP	University of Peloponnese
VR	Virtual Reality
